# The Clinical Laboratory

**Published:** 1912-11-02

**Authors:** 


### THE CLINICAL LABORATORY.

### I.—Rampant Pathology.

There has been during the last twenty years a tendency on the part of the teachers of the medical profession to lay increased stress on the importance of a thorough understanding of pathology by students and practitioners. While on the whole this tendency has been, undoubtedly, towards benefiting medical teaching, we are apt to overlook the fact that it has also materially added to the responsibilities of institutional workers. Perfect pathology demands perfect equipment. This entails considerable outlay which goes to swell expenses of upkeep, construction, and administration. Every modern hospital ward possesses a fully equipped ward laboratory, as we pointed out recently in the articles devoted to the construction of the modern hospital (" The Making of a Modern Hospital: The Ward Laboratory," The Hospital, May 4 and 18, 1912). In addition the institution, to be considered up to date, must have its large departmental laboratory attached to the pathological block, where experiments and researches such as cannot be carried on in the ward laboratory may be completed. Further, there should be an equally well installed chemical and physiological laboratory, with a bacteriological laboratory as adjunct. All this modern medicine demands, and it is a demand that administrators will have to face not only in hospitals which have teaching schools attached to them, but in purely charitable institutions. The reason is, of course, obvious. As we have before shown, modern methods of diagnosis and treatment rest largely on a pathological or pseudo-pathological basis. The student must be thoroughly acquainted with the views on which a particular line of diagnosis and palliative or curative treatment rests; he must be familiar later on when he comes to put his teaching into practice with the pathological principles that underlie the course which he proposes to take in the interests of his patients. All this is self-evident. But while admitting its obviousness, the administrative worker may be pardoned if he stops for a moment and asks himself the question, " Are we not favouring pathology to the detriment of purely clinical study? Is it well, in other words, that we should place the laboratory first and the ward or the bedside second? "

At first sight the rights of the case seem altogether to be on the side of the pathologists. They insist, with increasing vehemence, on exploiting the pathological side; they complain, as Dr. Flexner does in his report to the Carnegie Trustees, that too little attention is paid to the claims of the laboratory. They point out that most of the advances made in medicine since the introduction of anæsthetics have been the direct or indirect outcome of preliminary investigations patiently pursued in the laboratory, of suggestions made and tested by chemists, physiologists, bacteriologists, and pathologists. There can be no doubt that this is a strong argument, but it needs to be urged with care. The mass of medical students go, in after life, to attend patients in circumstances which do not admit of the medical man himself carrying out the elaborate technique involved in pathological examinations and chemical analyses. There must always remain a regiment of skilled and trained men who are prepared to undertake these investigations and give the practitioner the benefit of their expert knowledge. In practice this is already the case. The medical man, in the case of sore throat, takes a swab and sends it away to be tested for the presence of the Klebs-Loeffler bacillus. If he is well trained and able to diagnose diphtheria from the clinical evidence he will formulate his treatment without waiting to hear the bacteriologist's report. But it is a well-known fact that as we come to depend more and more on pathological methods of diagnosis we lose something of the power to diagnose on signs and symptoms. Contrast the teaching of a young physician, whose knowledge is largely derived from the laboratory and from books, with that of an experienced colleague, whose information comes from actual clinical observation, and note the immense difference in points of detail. The former insists, necessarily, on the importance of accuracy: he places the disease first of the symptom group; his pathology and morbid anatomy are almost faultless; but when it comes to treatment there is often a marked sense of being at a disadvantage. The latter, on the contrary, clears his pathology away in a few sentences: the primary importance, to him, is the treatment, the prognosis, and the symptomatology. It was Sir Dyce Duckworth, we think, who affirmed that the modern medical student is not properly taught how to satisfy the psychic wants of his patients. We have gained from chemotherapy a host of new drugs; we have lost the art of compounding our mixtures so that they shall not only be efficacious, but palatable, and by being palatable doubly efficacious. Similarly with regard to diagnosis. The experienced surgeon, familiar with the clinical signs, is a far better judge of the malignancy or innocency of a tongue tumour than the pathologically trained man who hesitates to give an opinion before he has had an opportunity of seeing a stained section of tumour tissue. What is more, the basis of pathology on which the latter grounds all his knowledge leads him often to be a blind adherent to one or other view that fulfils certain postulates which he has come to regard as axiomatic. Medicine today is full of fads, most of which are founded on what is taken to be pathological fact. One surgeon sees in every condition the baneful influence of intestinal sepsis; another, a physician this time, pins the physical welfare and moral comfort of humanity down to the avoidance of xanthin-containing food products. Both fashion their treatment along lines which agree with their own particular views, and both have followers whose Korans are the special articles which these gentlemen from time to time publish. It is not that the profession, as a whole, accepts either view any more than it accepts the tenets of the school of Hahnemann or Schrott, but the basis of pathology on which both appear to rest gives them a certain *cachet* which is respected even by those who totally deny the deductions drawn from the facts.

To the administrative worker the problem should present but little real difficulty. We may take it for granted that the objections outlined above can all be satisfactorily answered by those who favour the pathological method. For instance, it is better that the diagnosis of malignancy, seeing that it is of such tremendous and vital importance to all concerned, should rest on the clear finding of the microscope rather than that it should be settled by the empiricism of the experienced clinician. The clinician, however able, is fallible; the microscope is exact, and the only fallacy possible lies in the interpretation of what it shows. It is better, too, that a patient should be subjected to a prolonged preliminary examination, in which his stomach contents, his fæces, his food, and his urine are carefully analysed than that he should be operated upon for a possible cancer of the pylorus which turns out to be gastric atony relievable by medical means. Where it is a question of pure diagnosis the hospital cannot and ought not to afford to omit to equip itself in accordance with the demands of modern medical science, however great the outlay and however, at present, incommensurate the profit may seem. But it is wholly different when it comes to teaching, to research pure and simple, and to spending huge sums in equipping perfect laboratories for the needs of a few enthusiasts. The sphere of such investigators is altogether different from that of the hospital staff. Their work lies in another direction, and while they may, and should be, permitted to utilise the material of the hospital, so long as such utilisation is not directly or indirectly prejudicial to the interests of the patient, it is bad policy to allow their claims, as teachers or investigators, to supersede those of the institution wherein they work. It must be insisted upon that the hospital exists for the patients, not the patients for the hospital, and medical men attached to our charitable institutions, in their own interests, should be the first to admit the justice of the claim that the patients' interests are paramount. In practice it is often difficult to draw the line with any degree of sharpness, nor, indeed, is it desirable to do so. But it would be well if members of the committee took an active personal interest in the research work that is being carried on in their institutions and satisfied themselves that such investigation does not exceed the limits beyond which it should not progress. It may be said that it is wholly impossible that a layman can form any opinion on this matter, but we venture to think that the statistical data which the committeeman can always obtain affords a reasonable basis on which to formulate an opinion, while the medical members of a board can always be relied upon to clear up points that seem obscure. For example, if it is found that the drug bill of the analytical laboratory is unduly large—as when the director engages in private analysis work—it is comparatively easy to find out what is being done and to rectify any faults. Above all, when new laboratories are proposed it must be the duty of those responsible for their construction and upkeep carefully to consider whether they are not planned on a scale far beyond institutional requirements. In all these matters the help and advice of disinterested outside experts will be valuable, and the appointment of a trained assessor will be of great benefit to the hospital in coming ultimately to a decision.

